# Neurological Manifestations of X-Linked Ichthyosis: Case Report and Review of the Literature

**DOI:** 10.1155/2017/9086408

**Published:** 2017-08-13

**Authors:** William S. Baek, Umut Aypar

**Affiliations:** ^1^Parkside Medical Group, 1310 San Bernardino Rd, Suite 102, Upland, CA 91786, USA; ^2^Division of Laboratory Genetics, Department of Laboratory Medicine & Pathology, Mayo Clinic, 200 First Street SW, Rochester, MN 55905, USA

## Abstract

A 5-year-old boy presented with mild autism and attention-deficit hyperactivity disorder (ADHD). Chromosomal microarray demonstrated a 1.7 Mb deletion at Xp22.31, which was consistent with X-linked ichthyosis (XLI). Further exam revealed dry, scaly skin on his abdomen and pretibial areas. Patients with mutations involving solely the STS gene or the recurrent ~2 Mb deletion may present with ADHD, whereas those with larger deletions including the NLGN4 gene can present with both ADHD and autism. However, our patient presented with mild autism in addition to ADHD despite having only the recurrent deletion without loss of NLGN4. Such neurological manifestations of XLI warrant attention as practical targets of clinical management.

## 1. Introduction

X-linked ichthyosis (XLI) is an X-linked recessive disorder with a prevalence of approximately 1 : 4,000, demonstrating dry, scaly skin due to a deficiency of the enzyme steroid sulfatase [1]. While patients with mutations involving solely the STS gene or the recurrent ~2 Mb deletion can present with attention-deficit hyperactivity disorder (ADHD), those with larger deletions including neighboring genes such as neuroligin 4 (NLGN4) may present with autism in addition to ADHD [2, 3].

We present a case of XLI with ADHD and mild autism due to a recurrent ~2 Mb deletion which spared the NLGN4 gene.

## 2. Case Presentation

**Figure 1:**
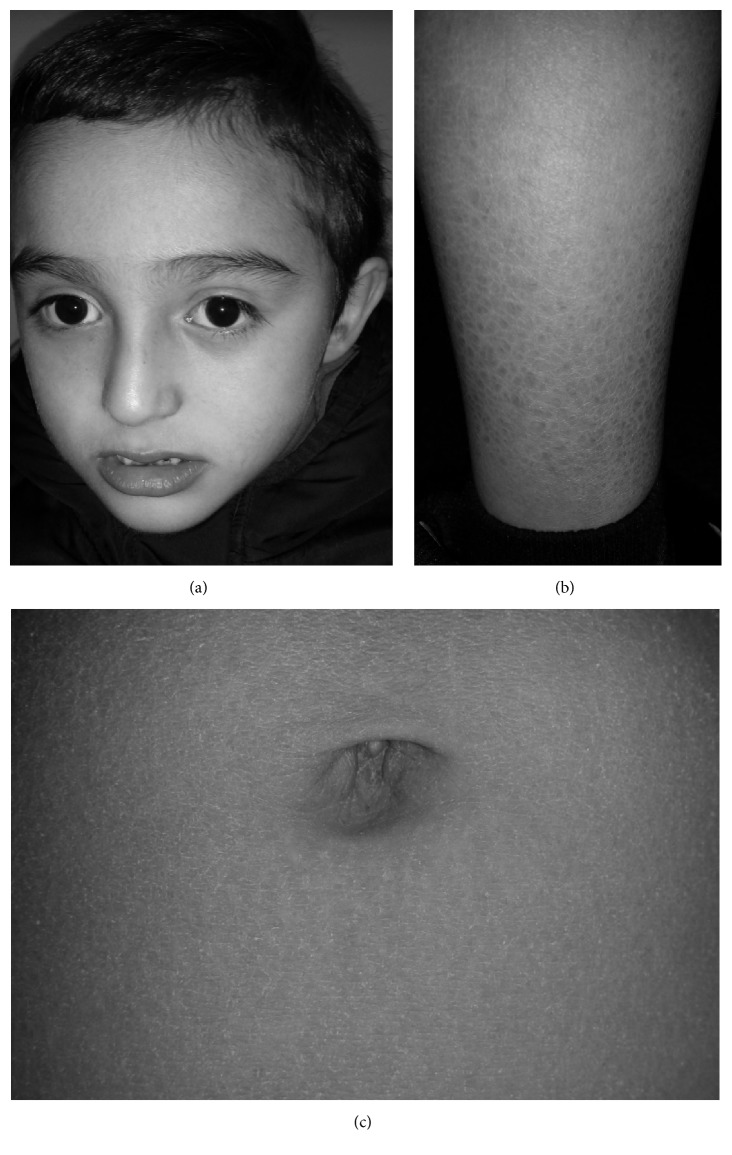
(a) Facial features include the high anterior hairline, a triangular face, a short chin, widely spaced eyes, and prominent nasal bridge. The dry, scaly skin was observed in (b) the pretibial areas and (c) the abdomen.

**Figure 2:**
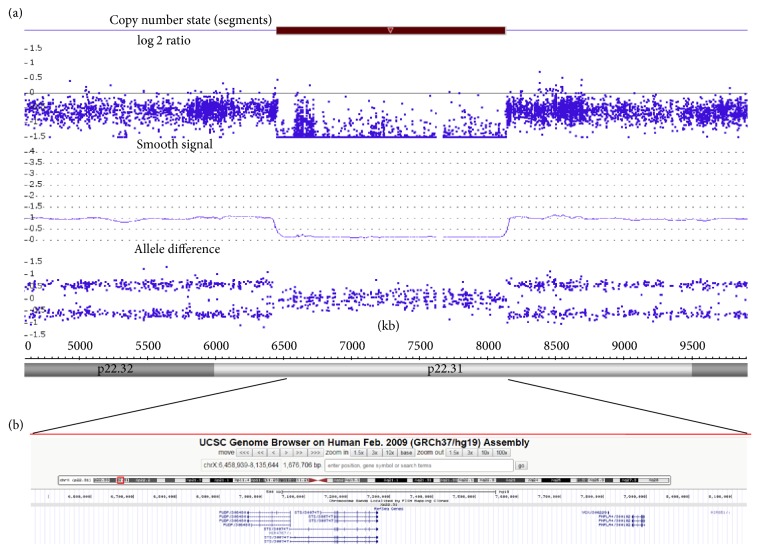
(a) Affymetrix CytoScan chromosomal microarray analysis detected a 1.7 Mb deletion at Xp22.31 (6,458,939–8,135,644, genome build hg 19) in our male patient. (b) UCSC genome browser image showing the Xp22.31 deletion. The deleted interval contains six known genes (PUDP, STS, MIR4767, VCX, PNPLA4, and MIR651).

A 5-year-old boy (43 inches, 50th percentile, and 42 lbs, 50th percentile) presented to Parkside Medical Group (Upland, CA) for a neurological evaluation. His mother remembered being told that he might have intellectual disability based on blood tests and an amniocentesis; however, these test results were not available. Otherwise, the pregnancy history and delivery were normal. He weighed 6 lbs 10 oz (20th percentile) at birth, walked at 14 months, and said “mama” and “papa” at 8-9 months. During early childhood he did not want to be around other children. He adhered to routines; for example, when his father would change his favorite TV channel he would become very angry. He spoke Spanish and some English. He attended special education at a preschool level and he could count up to 100. However, he could not focus in school. He was diagnosed with ADHD and mild autism based on the DSM V criteria by a child psychologist six months prior to the neurology visit. He was started on amphetamine-dextroamphetamine ER 10 mg daily, which improved his behavior both at home and at school. He started participating more in group activities. On exam, he was found to have a high anterior hairline, a triangular face, a short chin, widely spaced eyes, prominent nasal bridge, and conical teeth (Figure 1). There were no corneal opacities or preauricular scales. He was able to answer questions about his name, age, and what he liked, but there was a lack of reciprocity and eye contact. There were no tics. However, he was fidgety, could not wait his turn, was wandering about, was talking excessively in Spanish, and was interrupting his parents at times. Therefore, his Adderall XR was increased to 20 mg daily, but he became weepy and emotional; hence the dose was lowered back to 10 mg daily, but 7 months later the dose had to be increased back to 15 mg daily due to his persistent symptoms of ADHD. He was also treated for insomnia with guanfacine 0.5 mg at night. Of note, he had a history of asthma and was being treated with budesonide 0.5 mg/2 mL suspension and montelukast 4 mg daily. Family history was unremarkable. He has an 11-year-old healthy sister. There was no history of consanguinity. His EKG was normal. There was no history of seizures; EEG was not performed due to lack of cooperation. His lab results were all normal (cholesterol, TG, HDL, LDL, FSH, LH, TSH, ACTH, vitamin A, free retinol, and plac Lp PLA2) except for human growth hormone, which was elevated (1.6 ng/ml; normal range 0.01–0.97 ng/ml). He tested negative for fragile X syndrome with 29 CGG repeats. Chromosomal microarray (CMA), performed at the Mayo Clinic (Rochester, MN), demonstrated a 1.7 Mb deletion at Xp22.31 (Figure 2), which included 6 genes; STS, pseudouridine 5′-phosphatase (PUDP), microRNA 4767 (MIR4767), variable charge, X-linked (VCX), patatin-like phospholipase domain containing 4 (PNPLA4), and microRNA 651 (MIR651), which was consistent with the diagnosis of XLI [4, 5]. CMA was performed using both copy number and single-nucleotide polymorphism (SNP) probes on a whole genome array (CytoScan HD platform) (Affymetrix). The genome-wide functional resolution of this array is approximately 30 kb for deletions and 60 kb for duplications. The presence of dry, scaly skin on his abdomen and pretibial areas (Figure 1) was confirmed after receiving the results of the array. There were no signs of cryptorchidism. The patient's mother could not be tested due to lack of insurance coverage.

## 3. Discussion

Here we present a case of XLI with 1.7 Mb deletion at Xp22.31 which included the STS gene, as well as 5 other genes. In addition to the classic features of XLI of dry, scaly skin (Figure 1), he also presented with ADHD and mild autism. During the pregnancy, his mother recalls some blood tests that are not available but presumably they showed low or undetectable serum levels of unconjugated estriol, which can be seen in XLI.

XLI was first described by Sedgwick in 1863 [6]. XLI is X-linked recessive; therefore, it occurs almost exclusively in boys with a prevalence of approximately 1 : 4,000 [1]. It is due to a lack of the enzyme steroid sulfatase as a result of mutations or deletions of the STS gene, which is located in the distal part of the short arm of the X chromosome (Xp22.31) [7]. XLI presents with dry, scaly skin (ichthyosis), skin lesions, corneal opacities, and cryptorchidism [1]. Patients with mutations involving only the STS gene or the recurrent ~2 Mb deletion may present with ADHD, whereas those with larger deletions (involving not only STS or genes in the recurrent ~2 Mb deletion but also other neighboring genes such as NLGN4) may present with ADHD and/or autism [2, 3]. Since patients with only STS mutations or recurrent ~2 Mb deletions have not demonstrated any autistic features, it was considered that the loss of one or more of the neighboring genes within the larger deletions is contributing to autism [3].

In our patient, chromosomal microarray revealed the recurrent ~2 Mb deletion, which included the following genes: STS, PUDP, MIR4767, VCX, PNPLA4, and MIR651. The significance of PUDP, MIR4767, and MIR651 is uncertain as there is not much known about the biological role of these genes. PUDP encodes a member of the haloacid dehalogenase-like (HAD) hydrolase superfamily; however, the encoded protein has no known biological function [8]. There is no information available on MIR4767 and MIR651 with regard to their targets [8]. VCX (variable charge, X-linked) gene belongs to the VCX/Y gene family, all of which are expressed exclusively in male germ cells. While it is considered that the VCX/Y genes encode for small and highly charged nuclear proteins, their function is yet to be elucidated [8]. Although there have been conflicting reports on the association between VCX3A and an abnormal neurocognitive phenotype, no such correlation exists with the gene deleted in our patient (VCX) or with other gene family members (VCX2, VCX3B) [9, 10]. PNPLA4 encodes a member of the patatin-like family of phospholipases, which has both triacylglycerol lipase and transacylase activities. PNPLA4 may be involved in adipocyte triglyceride homeostasis [11]. It has also been implicated as a causal gene for autism [7]. STS belongs to the sulfatase family and hydrolyzes several 3-beta-hydroxysteroid sulfates, converting specifically the sulfated form of dehydroepiandrosterone, otherwise known as DHEA-S to DHEA [8]. These are both neurosteroids which exhibit effects on neurophysiological and behavioral processes. Lower levels of DHEA in the blood have been associated with ADHD phenotype [12]. This could explain the link between loss of STS gene function and ADHD in XLI patients. In addition, animal studies suggest that the STS gene may be implicated in processes involving attention [13].

Kent et al. [2] reported that, out of 25 male patients with either STS point mutations or recurrent ~2 Mb deletions, 40% fulfilled DSM-IV criteria for ADHD (compared to population rate; 3.6%), 80% of which were inattentive subtype. Five had fulfilled criteria for an autistic spectrum disorder or related language/communication disorders, and all of them had unusually large deletions of the STS gene with loss of the NLGN4 gene. Males with solely STS mutations or recurrent ~2 Mb deletions did not demonstrate autistic difficulties. Therefore, NLGN4 was suggested as a causative gene for autism in XLI patients with larger deletions.

In our patient, however, the recurrent ~2 Mb deletion (including STS, PUDP, MIR4767, VCX, PNPLA4, and MIR651 but sparing NLGN4) was sufficient to cause both mild autism and ADHD.

Our case report highlights the importance of a neurological evaluation in XLI due to the association with ADHD and autism, especially those with the recurrent ~2 Mb deletions and those with larger deletions including NLGN4. In addition, we suggest that clinical cytogenetics laboratories which report these deletions should indicate the risk for the neurological manifestations in their interpretation, in addition to the skin manifestations and other clinical features. In conclusion, we hope to bring awareness of the neurological manifestations of XLI among neurologists and geneticists (both laboratory and clinical).

## 4. Materials and Methods

Genomic DNA extraction and chromosomal microarray analysis were performed with the previously described methods [14]. Informed consent was obtained from the mother for the photographs.
